# Ribonucleotide reduction - horizontal transfer of a required function spans all three domains

**DOI:** 10.1186/1471-2148-10-383

**Published:** 2010-12-10

**Authors:** Daniel Lundin, Simonetta Gribaldo, Eduard Torrents, Britt-Marie Sjöberg, Anthony M Poole

**Affiliations:** 1 Department of Molecular Biology and Functional Genomics, Stockholm University, SE-106 91 Stockholm, Sweden; 2 Unite Biologie Moléculaire du Gène chez les Extremophiles (BMGE), Departement de Microbiologie, Institut Pasteur, Paris, France; 3 Institute for Bioengineering of Catalonia (IBEC), Scientific Park of Barcelona, Barcelona, Spain; 4 School of Biological Sciences, University of Canterbury, Christchurch 8140, New Zealand

## Abstract

**Background:**

Ribonucleotide reduction is the only *de novo *pathway for synthesis of deoxyribonucleotides, the building blocks of DNA. The reaction is catalysed by ribonucleotide reductases (RNRs), an ancient enzyme family comprised of three classes. Each class has distinct operational constraints, and are broadly distributed across organisms from all three domains, though few class I RNRs have been identified in archaeal genomes, and classes II and III likewise appear rare across eukaryotes. In this study, we examine whether this distribution is best explained by presence of all three classes in the Last Universal Common Ancestor (LUCA), or by horizontal gene transfer (HGT) of RNR genes. We also examine to what extent environmental factors may have impacted the distribution of RNR classes.

**Results:**

Our phylogenies show that the Last Eukaryotic Common Ancestor (LECA) possessed a class I RNR, but that the eukaryotic class I enzymes are not directly descended from class I RNRs in Archaea. Instead, our results indicate that archaeal class I RNR genes have been independently transferred from bacteria on two occasions. While LECA possessed a class I RNR, our trees indicate that this is ultimately bacterial in origin. We also find convincing evidence that eukaryotic class I RNR has been transferred to the Bacteroidetes, providing a stunning example of HGT from eukaryotes back to Bacteria. Based on our phylogenies and available genetic and genomic evidence, class II and III RNRs in eukaryotes also appear to have been transferred from Bacteria, with subsequent within-domain transfer between distantly-related eukaryotes. Under the three-domains hypothesis the RNR present in the last common ancestor of Archaea and eukaryotes appears, through a process of elimination, to have been a dimeric class II RNR, though limited sampling of eukaryotes precludes a firm conclusion as the data may be equally well accounted for by HGT.

**Conclusions:**

Horizontal gene transfer has clearly played an important role in the evolution of the RNR repertoire of organisms from all three domains of life. Our results clearly show that class I RNRs have spread to Archaea and eukaryotes via transfers from the bacterial domain, indicating that class I likely evolved in the Bacteria. However, against the backdrop of ongoing transfers, it is harder to establish whether class II or III RNRs were present in the LUCA, despite the fact that ribonucleotide reduction is an essential cellular reaction and was pivotal to the transition from RNA to DNA genomes. Instead, a general pattern of ongoing horizontal transmission emerges wherein environmental and enzyme operational constraints, especially the presence or absence of oxygen, are likely to be major determinants of the RNR repertoire of genomes.

## Background

Deoxyribonucleotides, the building blocks of DNA, are synthesised from their RNA counterparts, ribonucleotides, by reduction of the 2' hydroxyl group in a radical-based reaction catalysed by ribonucleotide reductases (RNRs). The reaction is the sole *de novo *pathway for synthesis of deoxyribonucleotides, and was likely pivotal to the transition from RNA- to DNA-based genomes [1-5]. RNR is thus essential for cellular life, as evident from the observation that all studied organisms code for at least one RNR (with the exception of a few intracellular pathogens that rely on deoxyribonucleotide salvage from their hosts) [6]. All extant RNRs are evolutionarily related, as evidenced by structural conservation of the catalytic core [7-10].

RNRs have been grouped into three broad classes based on the mechanism of radical generation, which is essential for the reaction. Class I RNRs generate a tyrosyl radical in a separate subunit (NrdB or NrdF in subclasses Ia and Ib respectively), from where it is transferred to the catalytic subunit (NrdA/NrdE) with each catalytic turnover. In the radical-generating subunit, the radical originates at an iron-oxygen centre, meaning class I RNRs are operational only under aerobic conditions. Class III RNRs also carry a stable radical, generated by an activase (NrdG), using S-adenosylmethionine (SAM) as cofactor. Radical generation requires cofactor cleavage whereupon the radical is transferred to the catalytic subunit (NrdD), and stored as a stable glycyl radical. Exposure to oxygen cleaves the enzyme at the glycyl radical; class III RNRs are hence operational under strictly anaerobic conditions [11,12]. Class II RNRs can be either monomers or dimers of NrdJ. Radical generation is via cleavage of deoxyadenosylcobalamin (AdoCbl, vitamin B_12 _coenzyme). No stable protein-based radical is formed, so radical generation and transfer to the active site occurs with each turnover. Class II RNRs operate independent of oxygen presence or absence, but the requirement for AdoCbl means they are cobalt-dependent. See table 1 for a comparison of RNR classes and [13-15] for reviews of RNR biochemistry, genetics and protein structure.

**Table 1:** The RNR classes

	Class I*	Class II	Class III
Operation	Aerobic	O_2 _independent, B_12 _dependent	Anaerobic SAM dependent

Structure	α_2_β_2_	α or α_2_	α_2_, activated by β_2_

Component names	α:NrdA or NrdE β: NrdB or NrdF	NrdJ	Enzyme proper: NrdD Activase: NrdG

Radical generating metal site	Fe-O-Fe	Co (in AdoCbl)	β: 4Fe-4S and AdoMet

Distribution	Common in bacteria universal in eukaryotes, rare in archaea	Common in bacteria and archaea, rare in eukaryotes	Common in bacteria and archaea, rare in eukaryotes

*Class I is traditionally subdivided into Ia (NrdA and NrdB) and Ib (NrdE and NrdF) respectively. Class Ib lacks activity regulation, and Ib operons contain two Ib-specific genes, a flavodoxin (NrdI) and a reductant (NrdH).

Genome analyses indicate that all three RNR classes are found across all three domains of life, but only a small minority of genomes carry genes for all three classes [6]. While all three RNR classes are widespread among bacteria, available genome data from archaea and eukaryotes reveal a patchy distribution. Class I RNRs are rare among archaea, but are present in all sequenced eukaryotic genomes (except two intracellular parasites). Classes II and III on the other hand, are common across archaeal genomes, with only a handful identified in eukaryote genomes. Furthermore, organisms encoding more than one class as well as more than one set of genes for a single class, are common [6]. While some cases of within-class RNR specialisation in DNA repair have been well studied [16], and a class I RNR subunit in mammals is under DNA damage control [17], the reaction biochemistries also dictate the existence of clear operational constraints between the classes.

Given the clear antiquity of ribonucleotide reduction, and the broad distribution of the three classes of ribonucleotide reductase, we sought to address whether this distribution is the result of ancient paralogy - possibly predating the Last Universal Common Ancestor (LUCA) - followed by differential loss among lineages, or whether the current distribution is instead the result of horizontal gene transfer (HGT). Under the latter model, receipt of additional RNR classes may extend the environmental conditions under which the recipient organism can sustain DNA replication (and hence reproduction).

To test these two hypotheses, we constructed phylogenetic trees using protein sequences from 73 archaea, 1297 bacteria, 162 eukaryotes and 188 viruses from all three classes of RNR. The resulting phylogenies are not exempt from the many problems that complicate phylogenetic estimation from ancient sequences (e.g. mutational saturation, varying rates of sequence change across the tree and long-branch attraction [18]), but, when combined with additional genetic evidence, nevertheless recover sufficient information to distinguish between the two hypotheses.

## Results

### Assessing RNR phylogenies for vertical versus horizontal transmission

**Figure 1:**
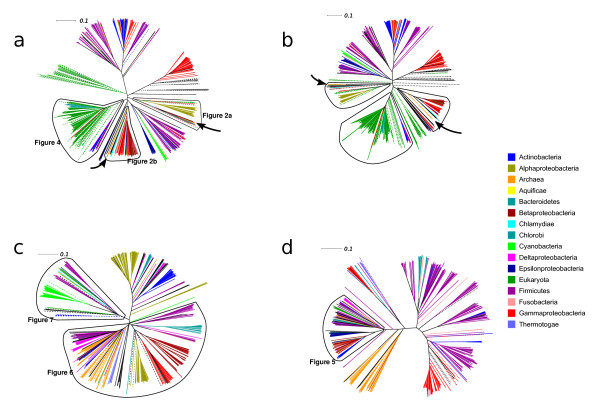
**Overview RNR phylogenies**. Unrooted BioNJ phylogenies from alignments of protein sequences of a) the catalytic subunit of class I RNR (NrdA and NrdE for subclass Ia and Ib respectively), b) the radical generating subunit of class I RNR (NrdB and NrdF for subclass Ia and Ib respectively), c) class II RNR (NrdJ) and d) the catalytic subunit of class III RNR (NrdD). Sequences were selected to cover the known sequence diversity. See inset colour legend for colours of the largest organismal groups. For black branches, see individual trees in additional file 1. Dashed branches are viruses; if the host range was known to us, viral sequences have the same colour as the host organism. Small arrows in a) and b) indicate the location of archaeal sequences. The marked parts of each tree were subjected to full maximum likelihood analyses, as indicated. All maximum likelihood trees are available in additional file 1; the trees from the marked areas of radical generating subunit of class I (NrdB, 1b) are only available in additional file 1.

To examine whether the evolutionary history of RNR genes reflects ancient paralogy with vertical descent or HGT, we performed phylogenetic analyses class by class (sequence divergence precluded analyses across classes). Figure 1 shows preliminary BioNJ trees for the class I catalytic (NrdA/E) and radical-generating (NrdB/F) subunits (figures 1a and 1b), the class II (NrdJ - figure 1c) and class III catalytic subunits (NrdD - figure 1d) respectively. The evolutionary distances spanned by known RNR distribution patterns are great, and we therefore expected to see poor resolution for the large number of sequences included in each tree. For ancient paralogy to apply, the three domains (Archaea, Bacteria and Eukarya) should be phylogenetically distinct - failure to see this pattern might either indicate insufficient phylogenetic signal across a given dataset, or that the data are better accounted for by horizontal transmission.

BioNJ trees of RNR subunits (figure 1) do not appear consistent with ancient paralogy under any plausible evolutionary model of the relationships between the three domains. A general pattern likely to be consistent with HGT is that major bacterial groups are divided across several disparate parts of the trees. For the pattern we observe to be the result of ancient paralogy followed by differential losses, a high number of RNRs would have had to have been present in the common ancestor of the various bacterial phyla. Further circumstantial evidence for the mobility of RNR genes is that RNRs are frequently encoded by HGT vectors such as plasmids, viruses, phage, and prophage (see RNRdb [6] and table 2).

**Table 2:** Examples of plasmid and prophage encoded RNR proteins

Domain	Organism	RNR proteins	Plasmid/prophage
Archaea	*Natronomonas pharaonis *DSM 2160	NrdA, NrdB, NrdR	PL131 (NC_007427)

Bacteria	*Acaryochloris marina *MBIC11017	NrdA, NrdB	pREB6 (NC_009931)

Bacteria	*Lactobacillus plantarum *L137	NrdD	pLTK13 (NC_011101)

Bacteria	*Lactococcus lactis*	NrdE, NrdF, NrdI	pGdh442 (NC_009435)

Bacteria	*Ralstonia eutropha *H16	NrdD, NrdG	pHG1 (NC_005241)

Bacteria	*Salmonella enterica *subsp. *enterica *serovar Typhi str. CT18	NrdA2, NrdB2	pHCM2 (NC_003385)

Bacteria	*Thermus thermophilus *HB8/HB27	NrdA, NrdB	pTT27 (NC_006462/NC_005838)

Bacteria	*Yersinia pestis *biovar Microtus str. 91001*	NrdA2, NrdB2	pMT1 (NC_005815)

Bacteria	*Bacillus subtilis *subsp. *subtilis *str. 168	NrdE2, NrdF2	prophage SPβ (NC_000964)

*There are several other examples of plasmid encoded class I RNR operons in *Yersinia *spp.

In order to investigate whether the distribution of RNRs across the three domains can be explained by HGT, we performed detailed investigations of a number of potential cases of interdomain HGT apparent from these preliminary analyses.

### Evidence for HGT of class I RNR from bacteria to archaea

**Figure 2:**
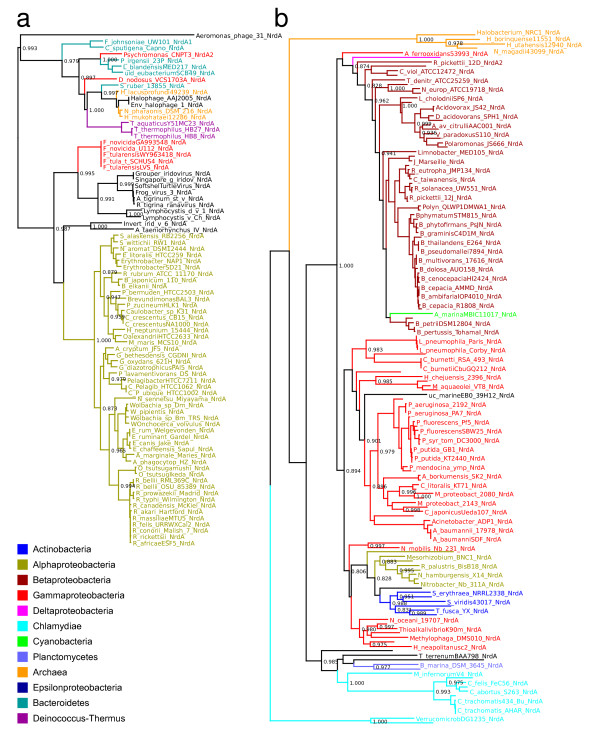
**Phylogenies of class I RNR catalytic subunits show independent transfers from bacteria to archaea**. PhyML UL3 phylogenies showing a) a probable bacterial origin of NrdA subunits from the archaea *Halomicrobium mukohataei *DSM 1228, *Halorubrum lacusprofundi *ATCC 49239 and *Natronomonas pharaonis *DSM 2160, and b) a probable bacterial origin of NrdA subunits from the archaea *Halobacterium *sp. NRC-1, *Halobacterium salinarum *R1, *Halogeometricum borinquense *DSM 11551, *Halorhabdus utahensis *DSM 12940 and *Natrialba magadii *ATCC 43099. Arrows point to the position of the archaeal sequences. Large top-level groups from the NCBI taxonomy have been colour-coded, see inset legend, and smaller groups are in black. Viruses are in italics. The tree is not formally rooted; the pseudoroot is placed so as to be consistent with the global phylogeny in Figure 1a.

**Figure 3:**
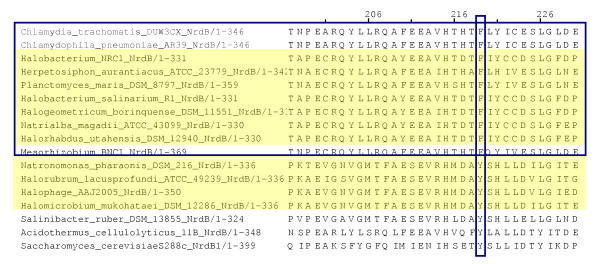
**Alignment of the radical generating subunit of subclass Ic RNR**. The proposed subclass Ic [77] (uppermost 10 sequences, in blue box) contains an unusual tyrosine to phenylalanine substitution (column in blue box). Subclass Ic hence does not harbour a stable protein radical [18,19]. Archaea are in a pale yellow box. From the alignment, it is apparent that a group of archaea (*Halobacterium *sp., *H. salinarium*, *H. borinquense*, *H. utahensis *and *N. magadii*) contains subclass Ic radical generating subunits, while another (*H. lacusprofundi*, *H. mukohataei*, *N. pharaonis *and *Halophage *AAJ2005) carry a normal tyrosyl radical-containing enzyme.

From our preliminary analysis of class I catalytic and radical-generating subunits (figures 1a and 1b) it was evident that the archaeal class I sequences form two well-separated groups (orange, indicated with arrows) interspersed within bacterial homologues (figures 1a and 1b). This is surprising given that all eight archaeal class I RNRs in RNRdb are from the Halobacteriaceae (Table 3). This pattern may therefore be indicative of one or more HGT events. To investigate this further, we generated maximum likelihood trees from the two clusters encompassing these archaeal sequences (indicated in orange in figure 1a). The resultant trees from both subunits (figure 2; see additional file 1 for NrdB/F trees), pseudorooted using the global topologies in figure 1 (see figure 2 legend), confirm this initial result, suggesting that at least one set of class I RNR genes were horizontally transferred after the diversification of the Halobacteriaceae. Additional evidence supporting transfer comes from an examination of the order of NrdA and NrdB genes in class I RNR operons: gene order is opposite in the two groups of archaeal sequences. The specific gene order is in both cases shared with bacterial nearest neighbours as identified from the trees in figure 2 (table 3). We also note that the small, radical-generating subunit (NrdB) in the group including *Halobacterium *sp. carries a substitution of the radical-bearing tyrosine to a phenylalanine (table 3 and figure 3). This variant is functional, as recently demonstrated for the class I RNR from *Chlamydia trachomatis *[18,19].

**Table 3:** Archaea and archaeal virus class I RNRs and nearest bacterial/phage neighbours*

	Organism	NCBI acc. nos	operon organisation	Comment
1	*Halomicrobium mukohataei *DSM 12286	NrdA: ZP_03875490	5'-nrdR-nrdB-nrdA-3'	
		NrdB: ZP_03875489		
		NrdJ: ZP_03874310		
		NrdR: ZP_03875488		

2	*Halorubrum lacusprofundi *ATCC 49239	NrdA: YP_002564381	5'-nrdR-nrdB-nrdA-3'	Class I on chromosome 2 (NC_012028, 0.5 Mb), class II on chromosome 1 (NC_012029, 2.7 Mb)
		NrdB: YP_002564382		
		NrdJ: YP_002567020		
		NrdR: YP_002564383		

3	*Natronomonas pharaonis *DSM 2160	NrdA: YP_327711	5'-nrdR-nrdB-nrdA-3'	Class I on plasmid (NC_007427), class II on main chromosome (NC_007426)
		NrdB: YP_327710		
		NrdJ: YP_327319		
		NrdR: YP_327708		

**4**	**Halophage AAJ-2005**	NrdA: ABB77922	5'-nrdB-nrdA-3'	
		NrdB: ABB77921		
		NrdJ: ABB77927		

**5**	***Salinibacter ruber *DSM 13855**	NrdA: YP_444285	5'-nrdB-nrdA-3'	The NrdA gene contains a group I intron
		NrdB: YP_444284		
		NrdJ: YP_444446		

6	*Halobacterium *sp. NRC-1	NrdA: NP_280998	5'-nrdA-nrdB-3'	Radical Y to F substitution
		NrdB: NP_280997		
		NrdJ: NP_280419		

7	*Halobacterium salinarum *R1	NrdA: YP_001690130	5'-nrdA-nrdB-3'	Radical Y to F substitution Both subunits identical to *Halobacterium *sp. NRC-1
		NrdB: YP_001690129		
		NrdJ: YP_001689532		

8	*Halogeometricum borinquense *DSM 11551	NrdA: ZP_04000564	5'-nrdR-nrdA-nrdB-3'	Radical Y to F substitution
		NrdB: ZP_04000565		
		NrdJ: ZP_03999548		
		NrdR: ZP_04000563		

9	*Halorhabdus utahensis *DSM 12940	NrdA: YP_003131237	5'-nrdR-nrdA-nrdB-3'	Radical Y to F substitution
		NrdB: YP_003131236		
		NrdD: YP_003130508		
		NrdG: YP_003130507		
		NrdJ: YP_003130199		
		NrdR: YP_003131238		

10	*Natrialba magadii *ATCC 43099	NrdA: ZP_03692957	5'-nrdR-nrdA-nrdB-3	Radical Y to F substitution
		NrdB: ZP_03692956		
		NrdJ: ZP_03694849		
		NrdR: ZP_03692958		

**11**	***Chlamydia trachomatis *D/UW-3/CX**	NrdA: NP_220348	5'-nrdR-nrdA-nrdB-3	Radical Y to F substitution
		NrdB: NP_220349		
		NrdR: NP_219916		

*Rows 1-3: archaeal class I RNRs transferred from halophilic bacteria, together with class I RNRs from the salt-tolerant bacterium *Salinibacter ruber *and a halophage (bold font, rows 4 & 5). Rows 6-10: archaeal class I RNRs that are similar to the sequences from the bacterium *Chlamydia trachomatis *(bold, row 11).

Several additional lines of evidence support two independent HGT events from bacteria to Halobacteriales. The group containing sequences from *Halomicrobium mukohataei, Halorubrum lacusprofundi *and *Natronomonas pharaonis *clusters with Halophage AAJ-2005 and the halophilic bacterium *Salinibacter ruber *13855 [20], with strong support in trees from both subunits (figure 2a and additional file 1). In addition to *S. ruber*, the nearest neighbours include other halophilic bacteria (*Psychromonas *sp. CNPT3 [21] and *Leeuwenhoekiella blandensis *MED217 [22]) but also the vertebrate pathogen *Dichelobacter nodosus *VCS1703a [23], the thermophilic *Thermus aquaticus *and *T. thermophilus *(both isolated from hot springs [24]) and the marine bacterium *Polaribacter irgensii *[25]. Phylogenetic proximity to halophilic bacteria adds credibility to transfer from bacteria to archaea since these species can plausibly come into contact in a hypersaline environment.

Closer examination of the data indicates several sequences are associated with vectors that could have facilitated transfer. In addition to Halophage AAJ-2005 (an archaeal virus), the class I RNR genes in *N. pharaonis *and in *T. thermophilus *are both encoded on plasmids [26] (table 2), likewise providing a possible vector for HGT. Interestingly, the class I genes in *Halorubrum lacusprofundi *are encoded on the second, smaller chromosome, whereas the class II RNR genes are encoded on the large chromosome (table 3). We can thus overall conclude that the group of archaeal class I RNR sequences from *Halorubrum lacusprofundi*, *N. pharaonis *and *Halomicrobium mukohataei *are likely the result of HGT from bacteria, most probably in a shared hypersaline environment.

The other group of halophilic archaeal class I RNRs (figure 2b, orange) - containing sequences from *Halobacterium *sp., *Halogeometricum borinquense*, *Halorhabdus utahensis *and *Natrialba magadi *- shows weaker positional support, but the combination of phylogenetic position, operon architecture (table 3) and a variant phenylalanine in the small subunit (NrdB) (figure 3) nevertheless indicates clear support for this group being distinct. While no vectors can be implicated as agents of transfer, and no sequences deriving from halophilic bacteria are immediate phylogenetic neighbours, to attribute the pattern we observe to vertical inheritance would require massive loss of class I RNR genes across archaea. This, and the fact that the NrdB genes carry an atypical substitution of the radical harbouring tyrosine to a non-radical harbouring phenylalanine, shared with *Chlamydiae *spp. and other bacteria that are relatively close to this group of archaeal sequences in our trees (figure 2b), suggests a second independent transfer of class I RNR genes from bacteria to archaea. These results are also consistent with other observations indicating extensive gene transfer events from bacteria into halophilic archaea [20,27,28]. In conclusion, the ancestor of extant archaea did not possess a class I RNR, and this class cannot be traced back to LUCA. Instead, it seems most likely that class I RNR evolved in bacteria.

### The LECA possessed a class I RNR of bacterial origin

**Figure 4:**
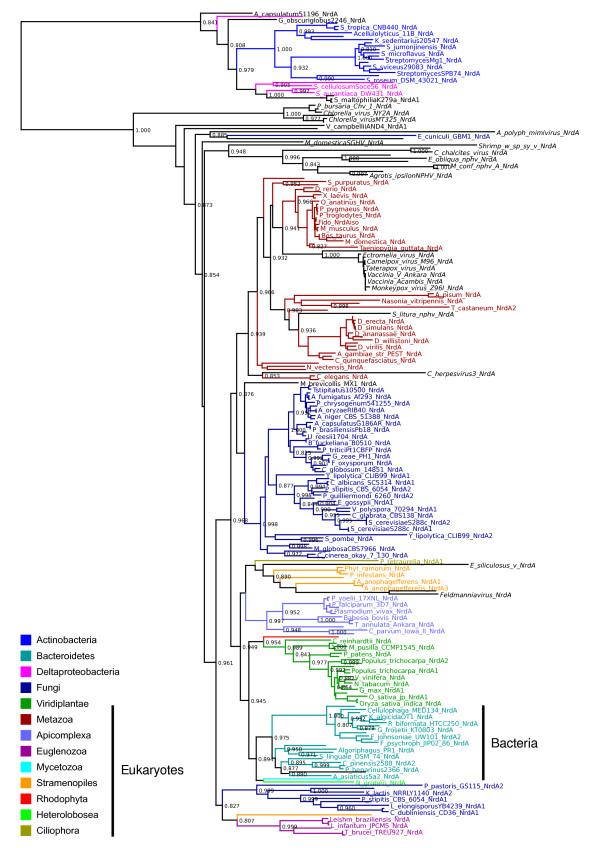
**Phylogeny of class I RNR catalytic subunit sequences shows a bacterial origin of eukaryote NrdA plus transfer from eukaryotes to bacteroidetes**. A PhyML UL3 phylogeny from an alignment of protein sequences of the catalytic subunit of class I RNR from eukaryote genomes, and a selection of closely related bacterial homologues. Large top-level groups from the NCBI taxonomy have been colour-coded, see inset legend with a bar indicating eukaryotic groups. Smaller groups are black and viruses are in italics. The bacterial sequences (mainly Bacteroidetes) we suggest were horizontally transferred from eukaryotes have been indicated with a bar and label. The tree is not formally rooted; the pseudoroot is placed so as to be consistent with the global phylogeny in Figure 1a.

Given the relatively recent bacterial origins established for archaeal class I RNRs, class I presence in eukaryotes cannot be explained by vertical inheritance from the common ancestor of eukaryotes and archaea. We have found class I RNRs in all completely sequenced eukaryote genomes we have knowledge of (August 2010) [6], which currently span five eukaryote supergroups. This suggests that the last eukaryotic common ancestor (LECA) may have possessed a class I RNR. In support of this, in trees derived from both class I subunits eukaryotic sequences form a clan, consistent with a single origin (figures 1 and 4; see additional file 1 for radical-generating (NrdB/F) subunit trees). Furthermore, phylogenies from both subunits (NrdA/E and NrdB/F) are broadly consistent across eukaryotes, and trees are likewise broadly congruent with expectations from single gene trees spanning eukaryotes. While there are unexpected positions for some groups, especially in the small subunit NrdB tree (see additional file 1), these largely relate to the relationships between eukaryotic supergroups; lack of resolution of the deepest branches of eukaryotes in our trees is hardly surprising given current difficulties in establishing a definitive eukaryote phylogeny from much larger datasets [29,30]. However, we do not observe any topologies that would strongly indicate horizontal gene transfer among eukaryotes, and we therefore tentatively conclude that LECA encoded a class I RNR.

It has previously been suggested that eukaryotic class I RNRs may have been transferred from mitochondria [3], and class I trees (figures 1 and 4) are consistent with a bacterial origin for eukaryotic class I RNR genes. A plausible sister group to eukaryotes, consisting mainly of actinobacterial sequences, can be identified in trees from both subunits (figure 4 and additional file 1). However, our trees do not provide sufficient resolution to establish a specific bacterial donor group. Moreover, there is no evidence that eukaryote class I RNRs are of alphaproteobacterial origin, which would be expected if the genes were a result of HGT from the protomitochondrial genome [31]. It thus seems likely that class I RNR entered the eukaryote lineage via HGT from a bacterium other than the protomitochondrion. However, with ongoing transfer of ribonucleotide reductases clearly occurring among bacteria, it is equally possible that any signal relating to the ultimate bacterial source has been erased by subsequent transfers; reliable detection of the originating lineage supposes that descendants of the donor have not themselves been subject to homologous gene displacement events.

### HGT of class I RNR from eukaryotes back into bacteria

Both NrdA and NrdB trees (figure 4 and additional file 1) also provide clear evidence for HGT of class I RNR in the opposite direction, from eukaryotes to bacteria. In both cases, there is strong phylogenetic support for the genes of the recipient bacterial group, predominantly composed of marine *Bacteroidetes *spp., being derived from within eukaryotes (figure 4 and additional file 1). While it is not possible to identify the exact partners associated with transfer, the known biology of members of the recipient group and their nearest eukaryote neighbours indicate a mechanistically and ecologically plausible scenario for association and transfer. Several species in the recipient group have a documented association with eukaryotes. For instance, *Algoriphagus *sp. is known to form colonies with the choanoflagellate *Proterospongia *sp. (for which no RNR sequence is available), *Psychroflexus torquis *was isolated from a sea-ice algal assemblage [32] and *Tenacibaculum *MED152 is a nitrogen-fixing bacterium associated with diatoms [33,34]. *Flavobacterium johnsoniae *UW101 is of particular interest since it encodes two class I operons; as seen in the NrdA trees, one is placed near the archaeal sequences in the tree (figure 2a), while the other groups with eukaryotes (figure 4). The nearest eukaryote neighbours in both NrdA and NrdB trees (figures 1 and 4; additional file 1) include oomycetes (*Phytophthora sojae *and *Aureococcus anophagefferens*) and *Naegleria gruberi *(an Excavate). Based on these characteristics, phagotrophy could plausibly have facilitated transfer, but would require prey bacteria to be resistant to digestion. While our analysis is unable to identify the specific eukaryote host-bacterium interaction responsible for the pattern we observe in the data, the observation that *Flavobacterium *spp. and *Amoebophilus asiactus *(both members of the Bacteroidetes) are digestion-resistant following engulfment by *Acanthamoeba *[35] is consistent with transfer by this route. It will therefore be interesting to establish whether gene transfer from host to resident bacteria - the opposite of the 'you are what you eat' ratchet [36] - is a significant pathway for gene acquisition.

### Eukaryotic class III RNRs and their activases are probably fused and have been transferred from bacteria

Class III RNRs are common among both bacteria and archaea [6]. Our overview tree in figure 1d (see also full tree in additional file 1), shows that archaeal and bacterial sequences are not intermixed, possibly indicating presence of class III RNR in the LUCA.

**Figure 5:**
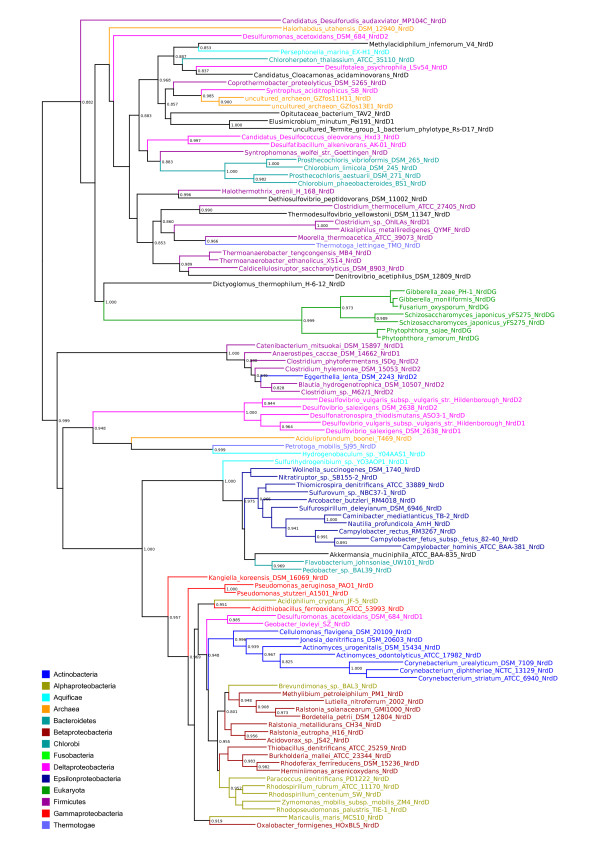
**Phylogeny of class III RNR from eukaryotes together with their most similar bacterial sequences**. A PhyML UL3 phylogeny from an alignment of NrdD protein sequences of the class III RNR enzyme from eukaryotic genomes and similar NrdD sequences from complete bacterial genomes. Only the NrdD part of the fused eukaryotic NrdDG proteins was used. The arrow indicates eukaryotic sequences. Large top-level groups from the NCBI taxonomy have been colour-coded, see inset legend, smaller groups are black. Viruses are in italics. The tree is not formally rooted; the pseudoroot is placed so as to be consistent with the global phylogeny in Figure 1d.

To date, only eight class III RNRs have been identified in eukaryote genomes [6], spanning fungi (*Schizosaccharomyces japonicus*, *Fusarium oxysporum, Gibberella moniliformis, Gibberella zeae*, *Nectria haematococca*), and stramenopiles (*Phytophtora ramorum*, *P. sojae *and *P. capsisi*). This might be due to limited sequencing of eukaryote genomes, or an indicator of HGT from either bacteria or archaea. Phylogenetic trees generated from NrdD sequences reveal that the eukaryote sequences form a well-supported clan (0.999 SH-like support - see methods), falling well within the bacteria, suggesting a single transfer into eukaryotes from a bacterial source (figures 1d and 5). For class III RNR to have been present in the LUCA would require, under the three domains scenario, that class III was present in the ancestor of archaea and eukaryotes and subsequently replaced in the lineage leading to LECA by HGT from a bacterial source. Further supporting a bacterial origin for eukaryotic class III RNRs, we note that the handful of archaeal sequences in the subtree shown in figure 5 are best accounted for by independent HGT events from bacteria, not vertical descent from the archaeal-eukaryote common ancestor.

Remarkably, closer inspection of the eukaryote class III operons reveals that, at the sequence level, NrdD and NrdG subunits are coded by a single open reading frame in all cases except in *S. japonicus *where there are two *nrdD *genes, one fused as per all other eukaryotes, and one singleton *nrdD*. HGT of fused proteins has been reported previously [37,38], and the putative NrdDG fusion raises the possibility that subunit fusion facilitates successful fixation of transferred prokaryotic operons in eukaryotes by permitting expression of distal genes in operons for which eukaryotic promoter sequences and translation initiation sites will be unavailable.

The existence of a eukaryotic NrdDG clan also suggests a single bacterial to eukaryote transfer with subsequent spread among eukaryotes. This is supported by the distribution of eukaryote NrdDG in two ways. First, this gene fusion is only sporadically present among fungi, being restricted to two genera. Notably, within the *Schizosaccharomyces *genus, only *S. japonicus *carries an NrdDG homologue - neither *S. pombe *nor *S. octosporus *genomes carry detectable homologues. Second, the only identifiable homologues outside fungi are in oomycetes *(P. ramorum *and *P. sojae*). This is of particular interest in that oomycetes have a fungal-like lifestyle, but are constituents of the supergroup Chromalveolata, so are evolutionarily distant from fungi (which are members of the Opisthokonta). Phylogenomic evidence suggests that the fungal-like lifestyle may in part be the result of extensive transfer of genes to oomycetes from fungi [39]. Taken together, these observations suggest that class III RNRs have been transferred from a bacterial donor to eukaryotes, with subsequent transfer between fungi and oomycetes.

### HGT of class II RNR from bacteria to eukaryotes, and intradomain HGT among eukaryotes

**Figure 6:**
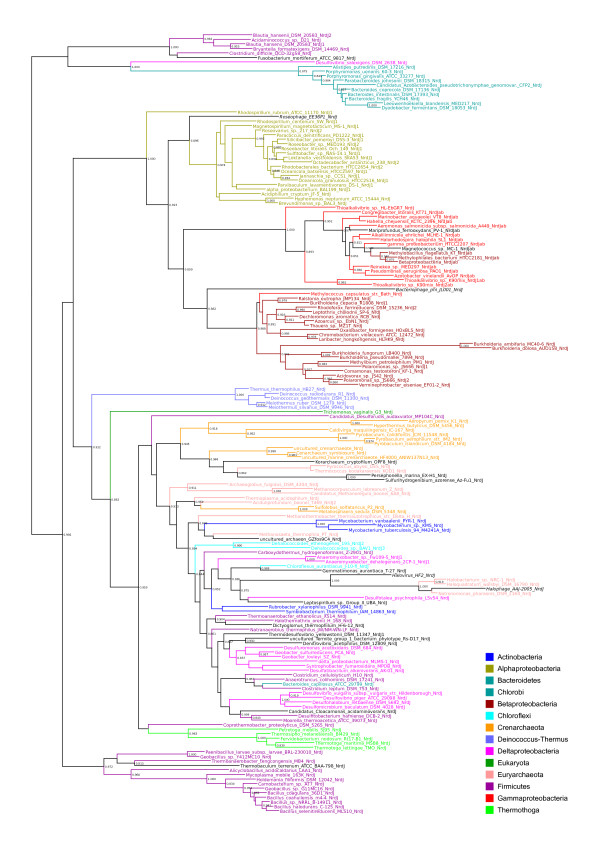
**Phylogeny of dimeric class II RNR**. A PhyML UL3 phylogeny from an alignment of protein sequences of dimeric class II RNR enzyme containing the eukaryote *T. vaginalis *(arrow). Large top-level groups from the NCBI taxonomy have been colour-coded see inset legend, smaller groups are black. Viruses are in italics. The tree is not formally rooted; the pseudoroot is placed so as to be consistent with the global phylogeny in Figure 1c.

The archaeal class II RNR sequences are all in the same part of our phylogenies (figures 1c and 6) although with several interspersed bacterial sequences. The topology is thus consistent with presence of a class II RNR in LUCA followed by a few HGT events between archaea and bacteria. However, certain bacterial groups - firmicutes (violet) and alphaproteobacteria (olive) in particular - are dispersed across the tree in a way that is hard to reconcile with a solely vertical pattern of descent.

**Figure 7:**
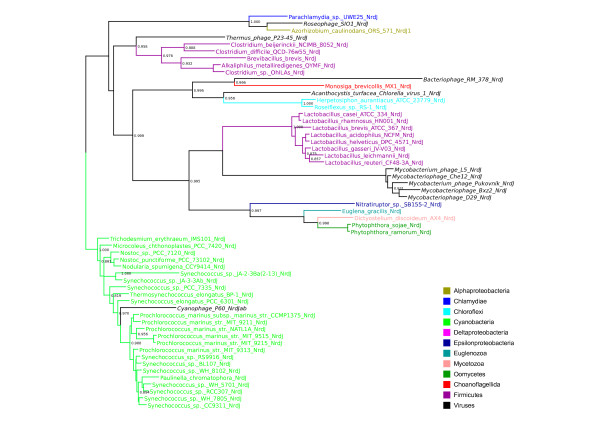
**Phylogeny of monomeric class II RNR from eukaryotes together with their most similar bacterial sequences**. A PhyML UL3 phylogeny from an alignment of protein sequences of monomeric class II RNR enzyme from eukaryotic genomes and genomes containing similar sequences. The arrows indicate eukaryotic sequences. Top level groups from the NCBI taxonomy have been colour-coded; viruses are in italics. The tree is not formally rooted; the pseudoroot is placed so as to be consistent with the global phylogeny in Figure 1c.

**Figure 8:**
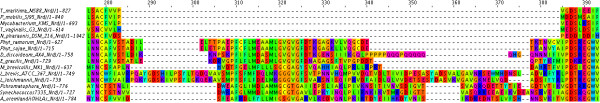
**Alignment of eukaryotic class II RNRs plus a few representative monomeric and dimeric prokaryotic class II RNRs**. The portion of the alignment shown here illustrates presence of the dimer interface-mimicking domain in monomeric enzymes and absence from dimeric class II RNR enzymes (top five sequences). The domain mimics the dimer interface, where the substrate specificity effector binds [9]. All eukaryotic proteins (*P. ramorum*, *P. sojae*, *D. discoideum*, *E. gracilis *and *M. brevicollis*) except the *T. vaginalis *sequence carry the insert, indicating they are monomeric. In support of this, the *P. ramorum *structure could not be modelled with the dimeric *T. maritima *structure using Swiss Model [78]. Nor could we model the *T. vaginalis *sequence with the *L. lactobacillus *structure, whereas the opposite combinations (*P. ramorum *with *L. lactobacillus *and *T. vaginalis *with *T. maritima*) successfully produce structure models (data not shown).

Similar to class III, class II RNRs are only sparsely distributed across sequenced eukaryotic genomes, though this distribution spans four eukaryote supergroups: Amoebozoa (*Dictyostelium discoideum*), Excavata (*Euglena gracilis *and *Trichomonas vaginalis*), Opisthokonta (*Monosiga brevicollis*) and Chromalveolata (*Phytophthora ramorum*, *P. infestans *and *P. sojae*). Such a broad distribution could indicate a deep eukaryotic ancestry for class II RNR. Notably, the class II RNR in *Euglena gracilis *has previously been suggested as a possible ancestral eukaryotic RNR [40]. If correct, this would suggest that a class II RNR was present in the LECA. If this was inherited vertically from the ancestor of archaea and eukaryotes, then the archaeal class II proteins should be closely related to the eukaryotic class II proteins. Alternatively, the sparse presence of class II RNRs among eukaryotes sequenced to date may be the result of HGT from bacteria. To test between these two possibilities, we generated phylogenies from NrdJ sequences (figures 1c, 6 and 7). Figure 1c shows, with strong support, that the eukaryotic class II RNRs can be divided into two phylogenetically distinct groups, consistent with at least one HGT event. One group (all species except *T. vaginalis*) is clearly derived from bacteria via HGT - all sequences in this group, except *M. brevicollis*, form a clan (0.997 SH-like support) - together with the epsilonproteobacterium *Nitratiruptor *sp. (isolated from a deep sea hydrothermal field [41]) - this clan is sister to a group containing the class II RNR from *Lactobacillus leichmannii *(figure 7). The structure of *L. leichmannii *class II RNR reveals it is a monomer [9], in contrast to the catalytic components of all other characterised RNRs, where the catalytic component forms a dimer [8,14]. At the dimer interface, effectors controlling the substrate specificity of the enzyme bind [7,8,10], but the monomeric *L. leichmannii *class II RNR instead contains a domain that mimics the dimer interface [9]. Our phylogeny is in broad agreement with previous studies where monomeric and dimeric sequences form separate groups [6,40]. An alignment of eukaryotic NrdJ sequences together with the sequences for the two structurally solved NrdJs (monomeric *L. leichmannii *and dimeric *T. maritima *respectively), and a handful of other putatively monomeric and dimeric NrdJ sequences, indicates that all eukaryotic NrdJ sequences, bar that from *T. vaginalis*, have the insert particular to monomeric NrdJs (figure 8).

The class II RNR from *M. brevicollis *also appears monomeric (figure 8), but does not belong to the same clan as the other monomeric eukaryotic sequences, suggesting this sequence may be the result of a second independent transfer. Branch lengths in the monomeric part of the tree are long however and the *M. brevicollis *sequence is extensively diverged from its nearest neighbours (Figure 7). This can lead to artefacts in phylogenetic reconstruction, especially long-branch attraction [42,43]. We performed successive removal of groups of sequences to analyse effects on topology, but failed to detect any artefact (data not shown). We therefore conclude that the presence of monomeric class II RNRs in eukaryotes is due to HGT from bacteria on at least one occasion. Establishing whether monomeric class II RNRs in eukaryotes originate from two independent transfer events must await the identification of additional eukaryote class II sequences.

While the monomeric class II RNRs in eukaryotes have entered this domain via HGT, what can be said of the evolutionary history of the *T. vaginalis *class II RNR? As all archaeal proteins are dimeric and group within a distinct clade (figure 1c), one possibility is that the *T. vaginalis *sequence has been inherited vertically from the archaeal/eukaryal ancestor. The tree indicates *T. vaginalis *is close to archaeal sequences (figure 6), but two complicating factors are evident. First, the branch leading to *T. vaginalis *is extremely long, and, second, the clan to which *T. vaginalis *is basal consists of archaea interspersed with bacteria (indicative of interdomain transfers), so there is no direct relationship with archaea as predicted under vertical inheritance. The extensive sequence divergence between *T. vaginalis *and archaeal class II sequences, plus the fact that no other dimeric class II sequences are known from eukaryotes, makes it difficult to conclude on current data whether the *T. vaginalis *class II sequence in our tree (figure 6) has a horizontal or vertical evolutionary history.

### Evaluating phylogenetic evidence for RNR gene mobility

A phylogenetic approach to identification of HGT can effectively identify transfer events between distantly related organisms since species incongruence is readily recognisable. We have identified a number of interdomain transfers on this basis, but establishing a direct donor-recipient relationship is often difficult because of limited sequence sampling, and conclusions can only be drawn from the resulting phylogenies. Following an interdomain transfer, spread of a gene via subsequent intradomain transfer is most readily identified when the later transfers are between distantly related species. However, phylogenetic signal may not in itself be sufficient to establish a transfer event since artefacts may also give a misleading signal (e.g. [44]). It has also been pointed out that the identification of putative transfer events is greatly strengthened by ecological plausibility [45,46], which in simple terms means that the candidate organisms should at least have overlapping environmental ranges, thus enabling interaction. In the preceding sections, we attempted to address ecological plausibility despite uncertainty regarding identification of donor and recipient. To address RNR mobility in more general terms, we therefore sought independent evidence for ongoing RNR gene mobility.

RNRdb contains a large number of RNR genes encoded in viral genomes [6], and, using the database, we identified several cases of plasmid and prophage encoded RNRs among bacteria (table 2). These constitute evidence for association of RNR genes with mobile vectors, and integrated plasmid and phage provide direct evidence for transfer, thus contributing to the view that RNR genes are horizontally mobile. This type of genetic signature readily identifies recipients, and demonstrates transfer events, but in most cases it is not possible to identify donors. One interesting case where a probable donor-recipient relationship emerges is for the class I RNR genes among a group of aquatic vertebrate viruses (including Frog virus 3 and Grouper iridovirus). These are not closely related to eukaryote class I RNR sequences. In both NrdA and B subunit trees (see figure 2 and additional file 1) these viral sequences are nearest neighbours to *Francisella tularensis*, a bacterial species that includes both mammalian and fish pathogens [47]. Further evidence consistent with a direct relationship is that both the *Francisella *and the viral sequences share unusual features: in all these sequences, the predicted NrdA lacks the N-terminal ATP-cone and the NrdB appears to be a translational fusion with glutaredoxin as it carries a glutaredoxin-like CxxC sequence in its N-terminal region. However, while the *nrdAB *genes form an operon in *Francisella*, they are separated in the viral genomes. The latter pattern suggests either independent transfers of each gene, or subsequent viral genome rearrangement. Our interpretation of these data is that these aquatic vertebrate viruses likely picked up class I RNR genes from *Francisella*, possibly during a double viral/bacterial infection in an aquatic vertebrate.

## Discussion

### Ribonucleotide reductases and the nature of LUCA and of LECA

Ribonucleotide reduction is the sole pathway for *de novo *deoxyribonucleotide synthesis, and evolution of this reaction is thought to have played a crucial role in the transition from RNA to DNA as genetic material [1-5]. Given such antiquity, it is perhaps unexpected that there are three major classes of RNR, each with distinct mechanisms for radical generation [4]. It is now clear that the catalytic core of the three classes shares a common ancestry [7-10], illustrating that ribonucleotide reduction evolved only once. The work we present here illustrates that phylogenies cannot be reliably used to settle the debate concerning the relationship of these three contemporary classes to the ancestral RNR [3,5,48,49], because the emerging pattern is one of extensive interdomain transfer. It is significant that there are numerous bacterial lineages which carry all three classes, and this is likewise true for two eukaryotes and one archaeon. The results presented here demonstrate that this pattern cannot be attributed to vertical descent from some totipotent LUCA, and instead illustrate that the patterns we see can be attributed to the disconnect between the evolutionary history of reproduction and gene mobility. Current data do not obviously favour a class I RNR encoded by LUCA. This interpretation is consistent with speculation that this class evolved from class II (classes I and II exhibit some sequence similarity though too low for reliable phylogenetic analyses), following the rise of atmospheric oxygen [3,48]. While we favour this interpretation, we note that cryptic ancient losses can never be ruled out (and are not inconceivable given the RNR gene mobility documented here), and an early origin of class I is in fact plausible under the 'respiration early' hypothesis [50-52], and given plausibility of abiotic oxygen generation in the early oceans [53]. Having said that, both class II and class III phylogenies are arguably more readily reconciled with presence in LUCA, though the general pattern of frequent intradomain and occasional interdomain horizontal transfers is such that, on these data, it is not possible to definitively establish whether LUCA was DNA-based [54].

While caution is warranted in speculating on the deep antiquity of ribonucleotide reductases, there is perhaps cause for cautious optimism with regards to establishing the ancestral state for ribonucleotide reduction in LECA however. Our results suggest that LECA probably possessed a class I RNR, but, having said that, there is also evidence that this is ultimately bacterial in origin, deriving from an ancient HGT event pre-dating LECA. Current data precludes the presence of class I RNRs in the common ancestor of eukaryotes and archaea on account of there being good evidence that those instances of class I RNRs in archaea are independent transfers from bacteria. Extrapolating from the operational constraints of class I enzymes, the implication is that LECA was at minimum aerotolerant and could divide in the presence of intracellular oxygen, consistent with the placement of sterol biosynthesis (which is oxygen-dependent) in LECA [55]. That known eukaryote class III genes have clearly entered this domain via HGT from bacteria rules out a strictly anaerobic RNR in the eukaryote ancestor. Having said that, our analysis of class II enzymes cannot formally exclude the possibility that LECA possessed a class II enzyme. However, the data in support of this possibility are restricted to the dimeric class II RNR from *Trichomonas vaginalis*. No eukaryotes are known to synthesise AdoCbl, and B_12_-utilisation across eukaryotes appears limited [56]. On the basis of these observations, support for a class II RNR ancestral to LECA is currently weak. This creates an interesting dilemma: all cellular lineages require at least one RNR (barring a small number of intracellular pathogens which can garner deoxyribonucleotides via salvage from their host); the class I RNRs may be traceable to LECA, but are ultimately bacterial in origin, and the dimeric class II RNR in *T. vaginalis *may yet turn out to be the result of a transfer event. If true, this would indicate that any evidence for the nature of ribonucleotide reduction in the eukaryote stem lineage (i.e. pre-LECA) has been erased by HGT.

More generally, our results are compatible with a bacterial origin of eukaryotic aerobic respiration, but the class I phylogeny does not enable us to establish that the source is mitochondrial, as previously suggested [3]. While it is tempting to try and establish the specific donor, the mobility of RNR genes may well have long since erased any trace of a mitochondrial origin since detection of such a signal is reliant on there being no HGT of alphaproteobacterial class I RNRs subsequent to this event. Two processes are expected to contribute to erasure of a signal for the direct donor for ancient transfer events. The first is transfer of the gene of a closest bacterial relative to the ancestor into another lineage. The second is displacement of the original sequence via loss or gene displacement in the direct ancestor. While it is still formally possible that the class I RNR entered eukaryotes via the mitochondrion, the fact that we see numerous transfer events into eukaryotes suggests a more complex picture, such as that proposed by Lester et al. [57], where genes of bacterial origin in the eukaryote lineage have been transferred from multiple bacterial sources.

### An ecological view of RNR gene transfer

With evidence for a vertical trace being limited in the case of RNRs, it seems highly likely that transfer of this essential function is facilitated by the varying operational constraints of each class. One might rather trivially account for ongoing transfer by alluding to the distinct operational constraints, and it likewise seems possible that cases where all three classes are present in the same genome may be indicative of lineages capable of undergoing reproduction in diverse environmental conditions. However, in contrast to some cases of one-off ancient gene transfer (where an ancient transfer event is evident, but where subsequent transfers are either not observed, or apparently infrequent), the data we present hints at an ongoing process of transfer and loss. In particular, RNRdb reveals numerous instances of paralogy as well as cases where only a single subunit is apparently present in a genome. This makes sense in that, under conditions wherein one RNR gene set is expressed, another may not contribute to deoxyribonucleotide synthesis. For instance, under environmental conditions where class I RNR is required, expression of class III may well be superfluous (indeed, the reaction biochemistries indicate the enzymes have mutually exclusive operational conditions). Consequently, under environmental conditions where one is essential the other may be lost through mutation, leading to a corresponding reduction in environmental range. Our prediction is therefore that horizontally transferred RNRs have a facultative symbiotic relationship with the vertically-inherited genetic cohort; at least one RNR is essential, but individual sets of RNR genes risk being lost. Rather than viewing gene transfer solely in terms of receipt of beneficial genes by the recipient, it would therefore seem reasonable to consider the ecology of RNR genes also. There is no benefit to gene loss for the individual gene, and as a consequence, those RNRs carried by vectors have the greatest chance of persisting and spreading under the threat of continual loss. Such a view may also help explain apparent redundancy across genomes (i.e. where more than one set of genes for a given class is present in a cellular genome). Under ongoing transfer, such redundancy may simply indicate the lag between integration and loss of one set of homologues. An examination of RNRdb reveals that this is relatively common. In some cases there may well be a functional rationale for such redundancy (it is common to find genes for both class Ia and Ib in bacterial genomes), but in the absence of direct evidence for subfunctionalisation and selection on subclass paralogues, the null hypothesis should be that this represents functional redundancy.

The best indication that all three classes may be under selection in some lineages comes from *Pseudomonas aeruginosa*, which encodes all three RNR classes [58-60]. All three classes are expressed in *P. aeruginosa*, though at very different levels, and there are indications for different roles of class I and class II during different cell cycle phases. During aerobic exponential growth, the level of class I transcripts greatly exceeds that of class II RNR. When entering stationary phase this pattern is reversed as class I transcription decreases and class II transcription increases [59]. A possible explanation for this is that the oxygen independence of class II could be an advantage during fluctuating or diminishing oxygen levels, a circumstance that is arguably more likely during stationary phase than during the rapid growth experienced when available nutrients allow exponential growth. The high expression levels of class I RNR during exponential growth suggest that class I RNR is more effective than class II in the presence of oxygen. Such an interpretation is in line with expectations of lower requirements for deoxyribonucleotides for repair purposes during stationary phase. Another possibility, suggested by Torrents et al. [59], is that *P. aeruginosa *experiences a reduced oxygen transfer rate during stationary phase, and this could reduce cellular oxygen concentrations to a level suboptimal for class I RNR function. In summary, although the precise biology is not yet known, the presence of all three RNR classes in *P. aeruginosa*, *Phytophthora *(a eukaryote), the archaeon *Halorhabdus utahensis*, and diverse bacteria [6] hints at a possible selective advantage for organisms that encode a range of RNRs. Equally, these observations could be an artefact of genome sequences as snapshots - we note that, in contrast to other *Phytophthora *genomes, the recently published genome of *Phytophthora infestans *[61] does not carry genes for class III RNRs. We therefore caution against extrapolating lifestyle effects or possible selective explanations from RNR gene repertoire alone.

Gene duplication, followed by specialisation via subfunctionalisation is considered an important evolutionary route to genetic novelty [62,63]. However HGT also creates a situation similar to paralogy by the presence of multiple copies. While a paralogous pair of genes created by gene duplication are initially identical, this is not necessarily so for xenologues created by HGT. HGT may therefore play an important role in organismal or lineage survival since it is a means by which 'ecoparalogues' [64] may be acquired by an organism.

## Conclusions

While ribonucleotide reduction is clearly an ancient process, pivotal to the origin of DNA, we find no definitive phylogenetic support for class II and class III RNRs being present in LUCA. Our data indicate class I originated in the bacteria, having spread to eukaryotes and archaea via horizontal gene transfer. The timing of the origin of ribonucleotide reduction therefore remains uncertain. We have however found evidence of both intra- and interdomain transfer of RNR genes and conclude that ribonucleotide reduction, the essential function encoded by these genes, is a mobile trait owing to the differing operational constraints that enzymes from the three classes display. We predict that organismal range will to some extent be dependent upon which classes are present in the genome of the organism, and that these are maintained through ongoing gene transfer.

## Methods

### Sequence selection

RNR protein sequences were downloaded from the ribonucleotide reductase database (RNRdb) [6], in which all non-environmental (i.e. metagenomic) RNR protein sequences in public databases have been collated and annotated. Because the number of sequences in RNRdb far exceeds the number of informative sites in complete multiple sequence alignments, the analyses presented here are based on representative selections of sequences. To select sequences, BioNJ [65] trees covering the full diversity of RNR components were generated as an aid to detecting potentially interesting patterns in the data (figure 1). Some divergent sequences - mainly viral - were excluded where these were uninformative for hypothesis testing as such sequences reduced the number of well aligned sites that could be used for phylogenetic analyses. Maximum likelihood (ML) analyses of subsets of the sequence diversity of each RNR component were subsequently performed (see marked clans in figure 1). In the trees from class I components (NrdA and NrdB), virtually all known sequences from the respective clans in the BioNJ trees were included in ML analyses, barring a few highly divergent sequences. For the class II and III ML trees, highly similar sequences from closely related species were eliminated. Both sets of trees (full BioNJ and subset ML) were compared, and different subsets were reanalysed where necessary in order to check that patterns detected in ML trees were robust and not an artefact of sequence exclusion. In additional file 2 we have listed NCBI accession numbers for each analysis.

Sequences for each subunit from each class were analysed separately. This was necessary for two reasons. First, while catalytic subunits of the three RNR classes are homologous (as evidenced by structural similarities [7-10]), performing a joint phylogenetic analysis from sequence alignment data of all three RNR classes is not possible since, in contrast to structure, sequence similarities between the catalytic subunits from the three classes are insufficient for generating global phylogenies. Indeed, some sequences have essentially no sequence level similarities except for a few strictly conserved residues like the catalytically active cysteine. Secondly, in the case of classes I and III, concatenating RNR subunit sequences from the same class was not practical. The activase of class III is far too short to be informative, and is in some cases non-trivial to distinguish from other members of the radical SAM family. Furthermore, complex patterns of paralogy, especially for class I protein-coding genes, will cause the components to contain divergent evolutionary signals.

### Sequence analyses

Alignments of protein sequences were performed using Probcons 1.12 [66] and Promals3D [67]. Well-aligned sites were chosen manually prior to phylogenetic estimation.

Phylogenetic trees were estimated using RAxML 7.2.6 [68-70] and a version of PhyML 3.0 that implements recently developed mixture models [71,72]. All PhyML analyses used the UL3 amino acid substitution model [73] and gamma substitution rate correction with eight categories. In additional file 2 we have listed parameters and number of positions used for each analysis. All trees presented in this paper and in associated supplementary material are unrooted.

In addition to the BioNJ trees and ML trees presented here, we also generated trees using maximum parsimony, Bayesian and network methods, as implemented in SplitsTree [74]. These alternative methods did not yield trees inconsistent with the results presented here (data not shown).

Branch support in PhyML analyses was inferred using the SH-like likelihood ratio test [75]. Phylogenetic tree figures were prepared using Dendroscope [76] and Inkscape.

## Abbreviations

RNR: Ribonucleotide reductase; LECA: Last Eukaryotic Common Ancestor; LUCA: Last Universal Common Ancestor; HGT: Horizontal Gene Transfer

## Authors' contributions

DL, AMP, ET & BMS designed research; DL performed research; DL, SG, BMS & AMP analysed data; DL, BMS & AMP wrote the manuscript, with input from all authors. All authors read and approved the final version.

## Supplementary Material

Additional file 1 **All PhyML trees in Dendroscope format**. This file is a gzipped tar archive containing all maximum likelihood trees presented here plus three additional trees from the class I radical-generating subunit (NrdB/F), in Dendroscope format. Individual files can be opened and read using dendroscope [76], which is available for download from: http://www.dendroscope.org/. Files have long informative names in which individual parts are separated by periods. First comes the name of the protein, second the name of the sequence selection, third the name of the alignment program (probcons in all cases), fourth the name of the site selection (wa00 in all cases) and fifth the PhyML parameters. See additional file 2 for descriptions of trees. Click here for file

Additional file 2 **Analysis details and translation tables**. This is an OpenOffice spreadsheet containing details regarding all analyses. The first sheet contains number of sequences and positions, plus PhyML parameters for each tree. Sheets T01-T13 contain full names and NCBI accession numbers for names used in our trees. Click here for file
